# Ethanol Extract of Licorice Alleviates HFD-Induced Liver Fat Accumulation in Association with Modulation of Gut Microbiota and Intestinal Metabolites in Obesity Mice

**DOI:** 10.3390/nu14194180

**Published:** 2022-10-08

**Authors:** Fei Liu, Xin Tang, Bingyong Mao, Qiuxiang Zhang, Jianxin Zhao, Shumao Cui, Wei Chen

**Affiliations:** 1 State Key Laboratory of Food Science and Technology, Jiangnan University, Wuxi 214122, China; 2 School of Food Science and Technology, Jiangnan University, Wuxi 214122, China; 3 National Engineering Research Center for Functional Food, Jiangnan University, Wuxi 214122, China

**Keywords:** licorice, ethanol extract, high-fat diet, hepatic lipid accumulation, gut microbiota, pseudo-germ-free

## Abstract

As a traditional Chinese medicine, licorice is often used in functional foods for its health benefits. However, the role of gut microbiota in the efficacy of licorice has not yet been fully elucidated. We hypothesized that the involvement of intestinal flora may be a key link in licorice ethanol extract (LEE)-induced health benefits. The aim of this study was to investigate whether LEE improves hepatic lipid accumulation in obese mice fed a high-fat diet (HFD) and whether the gut microbiota plays a key role in LEE treatment. Male C57BL/6J mice were fed HFD for liver fat accumulation and then treated with LEE. The same experiments were later performed using pseudo-sterile mice to verify the importance of gut flora. Supplementation with LEE improved the obesity profile, lipid profile and liver fat accumulation in HFD mice. In addition, LEE treatment improved intestinal flora dysbiosis caused by HFD in mice, as evidenced by a decrease in the percentage of *Firmicutes/Bacteroidetes* and an increase in the abundance of known anti-obesity-related bacteria. However, LEE failed to exhibit a therapeutic effect in pseudo-sterile mice. The results of the cellular assay showed that glycyrrhetic acid (GA), the main conversion product of glycyrrhizin (GL), was more effective in reducing fat accumulation and intracellular TG content in hepatocytes compared to GL. In conclusion, our data suggest that LEE attenuates obesity and hepatic fat accumulation in HFD mice, which may be associated with modulating the composition of gut microbiota and the conversion of LLE by the intestinal flora.

## 1. Introduction

In recent years, the rapid increase in obesity worldwide has led to its evolution into a global disease. Furthermore, an increase in the morbidity and mortality associated with obesity has been observed resulting from complications caused by this disease, including diabetes, cardiovascular disease, and non-alcoholic fatty liver disease (NAFLD) [1]. Numerous studies have suggested that obesity results from excessive fat accumulation due to an energy intake that exceeds energy expenditure. Obesity, glucose intolerance, insulin resistance, inflammation, and NAFLD are associated with chronic high-fat and high-fructose diets [2,3]. Visceral obesity and insulin resistance play a role in the development and maintenance of hepatic inflammation, and NAFLD is defined by hepatocellular fat accumulation and inflammation, both of which are factors that may significantly worsen health [4,5]. 

Functional foods containing beneficial phytochemicals can improve physiological and pathological responses to obesity through a variety of mechanisms, including the direct regulation of gene expression for obesity-related traits in the host and the improvement of gut dysregulation [6,7]. Glycyrrhiza is the dried root and rhizome of *Glycyrrhiza uralensis Fisch*, which has a broad variety of biological activities in traditional Chinese medicine and has been shown to significantly improve blood glucose and lipids in humans [8,9], as well as exert a significant effect in reducing liver damage and regulating lipid metabolism [8,10,11,12]. Although large-scale research is lacking, small-scale studies have indicated that licorice may safely and efficiently improve hepatic lipid buildup and liver function when used in conjunction with a high-fat diet (HFD) [9,13]. However, the mechanism of action of licorice in the treatment of NAFLD has yet to be fully elucidated, and gut microbiota may be an important factor in licorice therapy for NAFLD. 

Triterpenoids (e.g., glycyrrhizin) and flavonoids (e.g., isoglycyrrhizin) are the main bioactive substances in licorice. Studies have shown that the oral bioavailability of glycyrrhizin is only 4.0%, and it is mainly absorbed after direct or stepwise hydrolysis of two molecules of glucuronic acid by intestinal microorganisms to produce the saponin element glycyrrhetic acid, which is then further converted and absorbed through the hepatic-intestinal cycle [14,15]. Similarly, glycyrrhetinic acid and isoglycyrrhetinic acid need to be transformed by the intestinal flora for better absorption into the blood. Glycyrrhizin derivatives have been found to reduce obesity by modifying the gut microbiota and the intestinal barrier [16]. A growing body of evidence suggests that a complex interaction takes place between plant active components and gut microbes. Phytochemical health benefits may be influenced by gut flora [17,18,19]. However, there is no clear evidence that gut flora deficiency affects licorice health efficacy, and we hypothesize that the gut flora may play an important role in the ability of licorice to treat metabolic disorders; however, experiments are needed to demonstrate this. 

In this study, we first determined that licorice alcoholic extract could alleviate HFD-induced liver lipid accumulation in mice, and then performed the same experiment using pseudo-sterile mice to test the hypothesis that intestinal flora plays an important role in the therapeutic efficacy of licorice alcohol extract. 

## 2. Materials and Methods

### 2.1. Licorice Ethanol Extract (LEE) Preparation

A mill (DFY-800; Wenling Linda Machinery Co., Ltd., Wenling, China) was used to grind 100 g of dried licorice (Beijing Tong Ren Tang Co., Ltd., Beijing, China) into a fine powder, which was then sieved through a 60-mesh sieve. Fine licorice powder was weighed (50 g) and extracted for 3 h at 25 °C with constant stirring. After filtering the mixture, the residue was extracted twice with 500 mL of 95% ethanol in the same manner. Finally, all the filtrates were combined and centrifuged at 1000× *g* for 5 min, and the supernatant was concentrated using a rotary evaporator (R-100, BUCHI Labortechnik AG, Flawil, Switzerland) to obtain the extract, which was designated as LEE. The extract was stored in a refrigerator (−20 °C, BCD-610W, BSH Electric Appliances Co., Ltd., Nanjing, China) and used within 5 weeks. 

### 2.2. Animal, Diets, and Experimental Design

The animal ethics committee at Jiangnan University, Wuxi, China, approved the experimental procedures. The experiment was conducted at the Jiangnan University Animal Facility. Male C57BL/6J mice (4 weeks old) were acquired from Vital River Laboratory Animal Technology Co., Ltd. (Shanghai, China) and housed in temperature-controlled conditions (23 ± 2 °C, 12-h light/dark cycle) with unrestricted access to food and drink. After a one-week acclimation period, the mice were randomly divided into three groups (*n* = 6 per group) as follows: the control group, the model group, and the experimental group. The control group was fed a low-fat diet (LFD; 10% fat diet) (TP 23300; Trophic Animal Feed High-Tech Co., Ltd., Nantong, China), while the model and experimental groups were fed a high-fat diet (HFD; 60% fat diet (TP 23302). A 4-week intervention treatment was performed after 12 weeks of feeding. The control and model mice were gavaged with saline and the experimental mice were gavaged with LEE. The mice in the second experiment received the same treatment, but the intestinal flora of the mice was destroyed by antimicrobials for one week prior to LEE treatment. The antimicrobials consisted of 6.25 mg/mL vancomycin, 12.5 mg/mL ampicillin, 12.5 mg/mL metronidazole, 12.5 mg/mL neomycin (Sangon Biotech (Shanghai) Co., Ltd., Shanghai, China), and 0.2 mL per mouse per day by gavage. The antimicrobial-treated mice (PG mice) were gavaged with antimicrobials in the morning, whereas the other mice were gavaged with saline. The control and model mice were gavaged with saline in the afternoon, whereas the experimental mice were treated with LEE. Weight and food consumption were measured on a weekly basis. Three days before they were euthanized, fresh fecal pellets from each mouse were collected and they were kept at −80 °C in sterile EP tubes for future studies. Mice were starved overnight with access to water before being euthanized. Carbon dioxide anesthesia and cervical dislocation were used to euthanize all animals. To collect serum, blood samples were taken through heart puncture and centrifuged at 4 °C. Epidydimal white adipose tissue (EpiWAT), interscapular brown adipose tissue (intBAT), liver, kidney, and colon were removed and preserved at −80 °C (thermo-994, Thermo Fisher SCIENTIFIC (Shanghai), Shanghai, China) for further analysis. In addition, for the histological study, a part of the liver, epididymal white adipose tissue, and interscapular brown adipose tissue were preserved in a fixative solution (G1101-500ML, Wuhan Servicebio Co., Ltd., Wuhan, China). 

### 2.3. Oral Glucose Tolerance Test (OGTT)

OGTT was performed at the end of treatment. Briefly, a 20% (M/V) glucose solution was prepared and an oral glucose dose of 2.0 g/kg body weight was administered by gavage after 8 h of fasting in mice. The glucose levels were evaluated using a glucometer (ACCU-CHEK Active; Roche, Basel, Germany) after blood samples were taken from the tail vein at six separate time periods (0, 15, 30, 60, 90, and 120 min). The area under the curve for the OGTT was calculated. 

### 2.4. Histological Analysis

Freshly obtained tissues were fixed in a fixative solution for 48 h before being dehydrated and embedded in paraffin. Liver and fat tissue blocks were cut into 6-μm slices and stained with hematoxylin and eosin (H&E) for morphological analysis. Frozen slices of mouse livers were used for Oil Red O histochemical staining. A slice scanner (Pannoramic MIDI, 3DHISTECH, Budapest, Hungary) was used to scan H&E and Oil Red O sections for histological observation. 

### 2.5. Biochemical Analysis, Endotoxemia and Cytokine Measurement

The serum lipid profile and liver function index, including alanine aminotransferase (ALT), aspartate aminotransferase (AST), alkaline phosphatase (ALP), cholinesterase (CHE), triglycerides (TG), total cholesterol (TC), low-density lipoprotein cholesterol (LDL-c), and high-density lipoprotein cholesterol (HDL-c), were assayed using an automatic biochemical analyzer (Mindrayn BS-480; Shenzhen Mindray Bio-Medical Electronics Co., Ltd, Shenzhen, China). ELISA kits were used to measure the levels of tumor necrosis factor-alpha (TNF-α), interleukin 6 (IL-6), and interleukin 10 (IL-10) in the liver (ProteinTech, Wuhan, China). The ELISA kit instructions indicate that the intra-assay and inter-assay coefficients of variation are less than 10 %.

### 2.6. Real-Time PCR Analysis

Total RNA was isolated from liver tissues using the TRIzol Total RNA Isolation Kit (B511321; Sangon Biotech Co., Ltd., Shanghai, China). According to the manufacturer’s instructions, the samples were diluted using Trizol, and total RNA was obtained after homogenization, separation, extraction, purification, adsorption, and elution. Next, we measured the concentration of the collected RNA, and a reverse transcription system was used to convert 1 μg of RNA into cDNA (R323; Vazyme Biotech Co., Ltd., Nanjing, China). Real-time PCR was performed using a BioRad-CFX384 Touch thermocycler (Bio-Rad, Hercules, CA, USA) and Universal SYBR qPCR Master Mix (Q711; Vazyme Biotech Co., Ltd., Nanjing, China). All samples were tested in quadruplicate. Glyceraldehyde-3-phosphate dehydrogenase (GAPDH) was used as a housekeeping gene and the data were standardized using the ΔΔCT method for each sample. The target genes included the lipid metabolism genes fatty acid synthase (FASN), acylglycerol lipase (MGL), hormone-sensitive lipase (HSL), and patatin-like phospholipase domain-containing protein 2 (ATGL). 

### 2.7. Measurement of Short-Chain Fatty Acids (SCFAs) Concentration in the Colon Digested

Short-chain fatty acids were extracted using ether according to a previously published method [20,21]. Briefly, the samples were diluted with a saturated NaCl solution, acidified with sulfuric acid, extracted, centrifuged and the supernatant was placed into the injection vial. Gas chromatography-mass spectrometry (GC-MS) was used to analyze the amounts of SCFAs in the colonic digesta [22]. 

### 2.8. Gut Microbiota Analysis

A previously reported approach was used to determine microbial community composition in fecal samples [22]. The OmicStudio tools (https://www.omicstudio.cn, accessed on 15 July 2022) were used to create a clustering correlation heatmap to evaluate coefficient values between feature genera abundance and hepatic fat accumulation-related parameters and to test their significance. 

### 2.9. Cell Experiment

Cytotoxicity assays for LEE, GA and GL were performed, and cell viability was determined by the CCK-8 kit. Referring to a previous method [23], the hepatocyte fat accumulation model was prepared by treating HepG2 cells with 1 mM free fatty acid (FFA) mixture (oleic/palmitic acid, 2:1) for 18 h. The appropriate administration concentrations were selected to intervene in FFA-induced HepG2 cells based on the results of cytotoxicity assays. CCK-8 and TG kits (Nanjing Jiancheng Bioengineering Institute, Nanjing, China) were used to detect cell activity and intracellular TG levels, respectively. The medium was removed and 0.1 mL of sterile water was added, after which it was frozen and thawed three times before being tested with the TG kit. Oil Red O staining was used to evaluate intracellular lipid accumulation, followed by dissolution of stained lipid droplets using isopropyl alcohol and the determination of absorbance at 490 nm to quantify lipid aggregation. 

### 2.10. Statistical Analysis

GraphPad Prism 7.0 was used for the statistical analysis of the data. The results of a Shapiro–Wilk normality test were used to ensure that the numbers were distributed normally. ANOVA with the Tukey’s test was used to evaluate whether there were any significant differences between the groups. The results were expressed as the mean ± standard deviation, and each independent experiment was performed with a minimum of six biological replicates [4]. The threshold for statistical significance was set at *p* < 0.05. 

## 3. Results

### 3.1. LEE Administration Attenuated the Obesity Characteristics of HFD Mice

**Figure 1:**
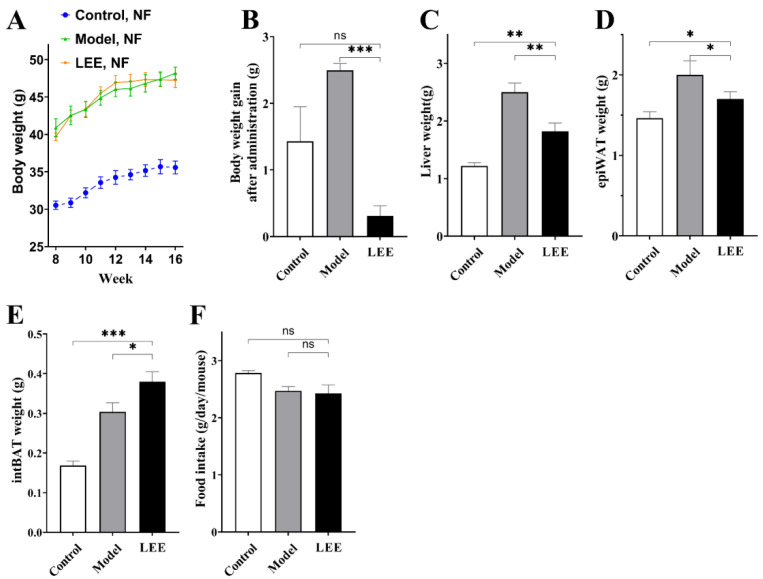
LEE relieves obesity symptoms in normal flora HFD mice. (**A**) Weekly body weight after intervention. (**B**) Body weight gain after administration. (**C**) Liver weight. (**D**) epiWAT weight. (**E**) intBAT weight. (**F**) Food intake. Data are expressed as the mean ± SEM (*n* = 6). * *p* < 0.05; ** *p* < 0.01; *** *p* < 0.001; ns, not significant.

Figure 1A shows the results of an 8-week study in which we examined the effects of LEE treatment on HFD mice weight, with steady increases in body weight in mice on LFD and HFD diets, and changes in body weight gain resulting from LEE intervention. After LEE administration, LEE mice showed a lower body weight gain than control and model mice, and LEE administration reduced the body weight increase generated by HFD feeding (*p* < 0.001; Figure 1B). Liver, epiWAT, and intBAT weights were significantly increased in HFD mice compared to those in LFD mice, whereas LEE intervention significantly reduced liver and epiWAT weights and further increased intBAT weight in HFD mice (*p* < 0.05; Figure 1C–E). There was no significant change in food consumption after LEE treatment (Figure 1F), suggesting that the effects of LEE on body weight and obesity were independent of the changes in food intake.

### 3.2. Effects of LEE on Serum Lipid Profile and Glucose Tolerance

**Figure 2:**
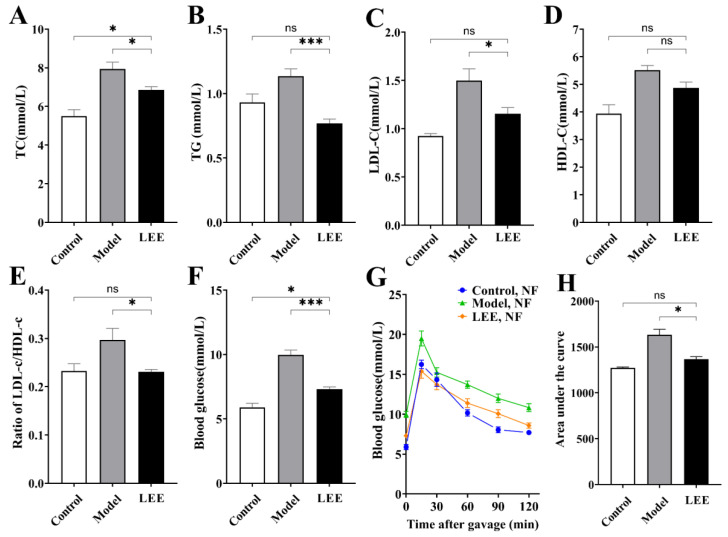
LEE improves dyslipidemia and glucose tolerance in HFD mice. Serum TC (**A**), TG (**B**), LDL-c (**C**), HDL-c (**D**), ratio of LDL-c/HDL-c (**E**), blood glucose (**F**), OGTT (**G**), and area under the OGTT curve (**H**). Data are expressed as the mean ± SEM (*n* = 6). * *p* < 0.05; *** *p* < 0.001; ns, not significant.

Since lipids and glucose are intimately linked to HFD-induced obesity, we measured the lipid and glucose parameters in HFD mice with or without LEE treatment. HFD intervention resulted in significant increases in blood lipid biochemical markers, including TC, TG, LDL-C, HDL-C, and LDL-C/HDL-C ratio, compared to those in the LFD group (Figure 2A–E). Unsurprisingly, these serum markers were reduced to varying degrees after LEE treatment, with values more similar to those of the LFD diet mice, indicating that LEE is effective in ameliorating dyslipidemia induced by a HFD. Additionally, a HFD significantly elevated fasting blood glucose levels and decreased glucose tolerance in mice. LEE treatment significantly reduced the effects of the HFD, as evidenced by the fact that the maximum blood glucose value after oral administration of 2.0 g/kg glucose and the blood glucose index after 120 min were significantly lower than those of the mice in the model group.

### 3.3. LEE Treatment Reduces HFD-Induced Liver Fat Accumulation and Damage

**Figure 3:**
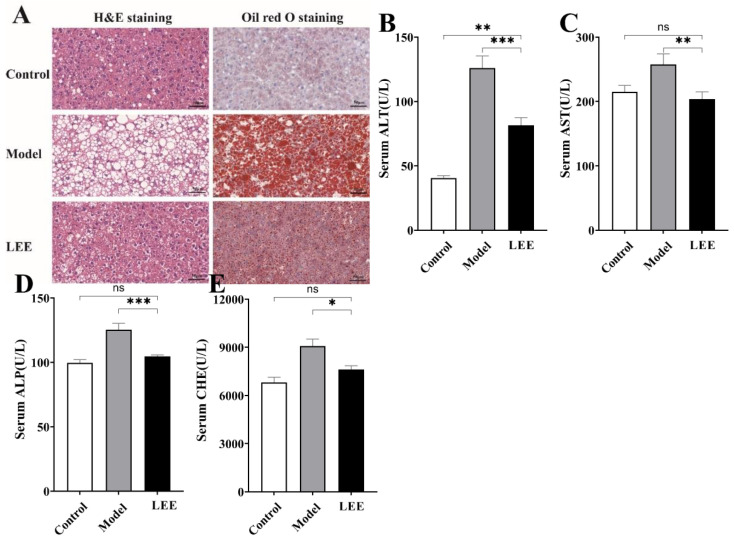
LEE administration alleviates HFD-induced liver steatosis and dysfunction in mice. (**A**) The staining of liver tissue sections (×400). Serum ALT (**B**), AST (**C**), ALP (**D**) and CHE (**E**) levels. Data are expressed as the mean ± SEM (*n* = 6). * *p* < 0.05; ** *p* < 0.01; *** *p* < 0.001; ns, not significant.

The liver is an important organ for metabolism. Long-term HFD can lead to the accumulation of lipids in the liver and a decline in liver function, which is a key signal for HFD-induced liver disease. The development of liver lesions was evaluated by means of liver histology. Histological analysis showed abnormal lipid accumulation and cellular morphology in the liver of HFD mice compared to those in the LFD mice, and LEE treatment significantly attenuated HFD-induced hepatic fat accumulation (Figure 3A). Model mice showed significant hepatocellular deformation, and both micro- and macro-vesicular fat accumulation with greater steatosis. LEE mice showed significant remission of liver lesions, as evidenced by the recovery of hepatocyte morphology, a significant reduction in micro-vesicular fat accumulation, and the disappearance of macrovesicular fat accumulation. Unsurprisingly, HFD causes liver damage in mice, including elevated serum ALT, AST, ALP and CHE levels, indicating that liver fat accumulation caused hepatocyte damage and inflammation. The content of liver function-related indexes in serum was significantly reduced by LEE intervention (*p* < 0.05; Figure 3B–E), suggesting that LEE has the ability to restore liver function damage caused by HFD.

### 3.4. LEE Treatment Changes the Composition of Gut Microbiota in HFD Mice

**Figure 4:**
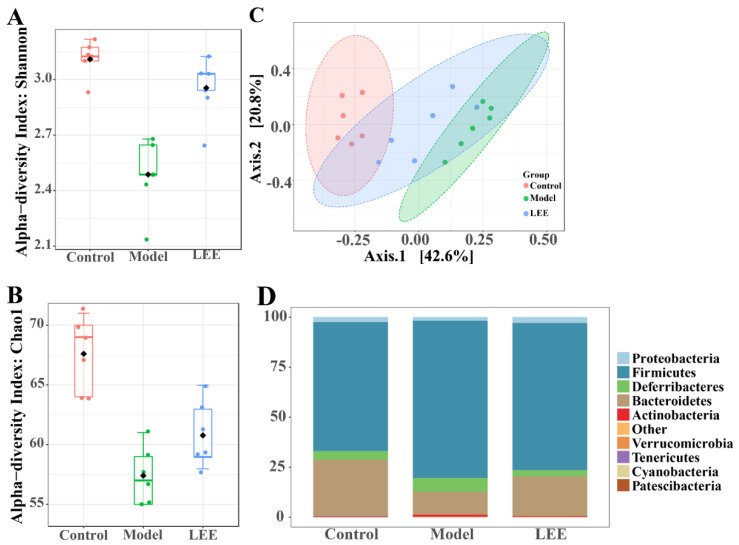
LEE alters the composition of intestinal flora in HFD mice. Shannon (**A**) and Chao1 (**B**) indexes indicate alpha diversity. Principal coordinates analysis of microbial taxa (**C**). Relative abundance at the phylum level (**D**). Data are expressed as the mean ± SEM (*n* = 6).

The intestinal flora composition of the mice was examined. The higher Shannon and Chao1 indices in the LEE mice than in the model mice (Figure 4A,B) revealed that LEE treatment successfully reversed the HFD-induced loss in alpha diversity. A principal coordinate analysis (PCoA) using the UniFrac distance indicated unique clustering of the microbiota composition among the various treatments (Figure 4C). The results showed that HFD mice clustered separately from LFD mice, which revealed that the intestinal microbial communities of HFD and LFD mice were considerably different. The LEE and control groups exhibited more overlap, indicating that LEE therapy had a significant impact on the conversion of gut microbial composition from HFD mice to LFD animals. As diet has an important impact on the composition of the gut microbiota, the relative abundance of the predominant taxa were examined by sequencing each community to identify alterations in the gut microbiota. The gut microbiota of normal mice was mostly composed of *Proteobacteria*, *Firmicutes*, *Deferribacteres*, and *Bacteroidetes*, according to the relative abundance histogram of intestinal flora at the phylum level, which is consistent with the results of earlier studies. The intestinal microbial structure was clearly altered by HFD treatment, with a considerable decrease in the relative abundance of *Proteobacteria* and a marked elevation in *Firmicutes* (Figure 4D), resulting in a significantly elevated F/B ratio. LEE administration reversed the structural proportion of the intestinal flora at the phylum level in HFD mice, especially *Firmicutes*, *Bacteroidetes* and *Deferribacteres*, and reduced the F/B ratio. Compared with the model mice, the structure of intestinal flora in LEE mice was more consistent with that in the control mice at the family level. 

**Figure 5:**
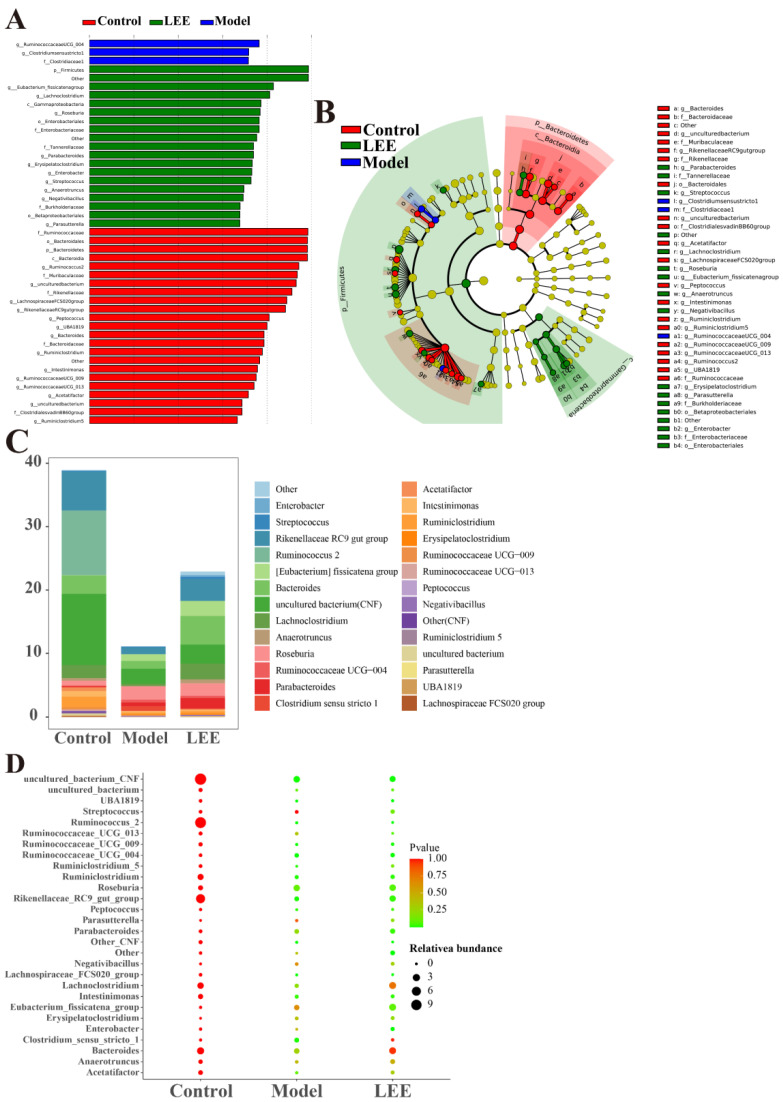
*LEfse* analyses of intestinal flora in control, model and LEE group. Histogram shows the LDA scores computed for features (genus level) differentially abundant between different groups (**A**). Cladograms representing the LDA effect size (*LEfse*) results for the control, model and LEE groups (**B**). Composition and abundance of the samples of *Enterobacteria* at discrepant genus (**C**). *p*-values and relative abundances of the microbiota markers at the genus level in the different groups. The *p*-values were calculated by comparison with those of the control group (**D**).

We used *LEfse* among control, model, and LEE mice (LDA > 3) to identify the particular phylotypes that were significantly regulated in response to HFD and LEE treatments (Figure 5A,B). The results revealed that 39 OTUs (24 genera, 3 phyla) were chosen as key nodes, with abundances considerably affected by HFD and LEE therapy. HFD administration altered the feature-level intestinal flora *Streptococcus* and *Negativibacillus*, which belong to *Firmicutes*. LEE administration led to 12 feature-level intestinal flora, which mainly belong to *Firmicutes*, *Proteobacteria* and *Bacteroidetes*. According to the *LEfse* analysis, microbiota markers at the feature-genera level were selected for further study. The feature genera level structure in the LEE group was more similar to that of the control group (Figure 5C,D), and the bubble diagram shows that after LEE treatment, the four feature genera level (*Lachnoclostridium*, *Clostridium sensu strieto 1*, *Bacteroides*, and *Anaerotruncus*) in the LEE group were similar to those in the control group, while the levels of four feature genera (*Streptococeus*, *Parasutterella*, *Negativibaeillus*, and *Eubacterium_fissicatena_group*) were more different from those of the control and model groups (Figure 5D). These findings revealed that LEE treatment changed the structure and composition of the gut microbiota in HFD mice.

### 3.5. Effect of LEE on Pseudo-Germ-Free Mice with HFD

**Figure 6:**
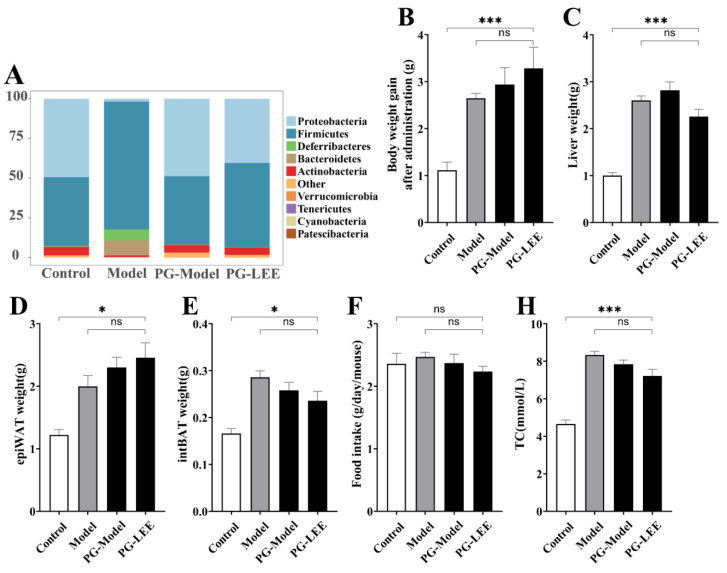

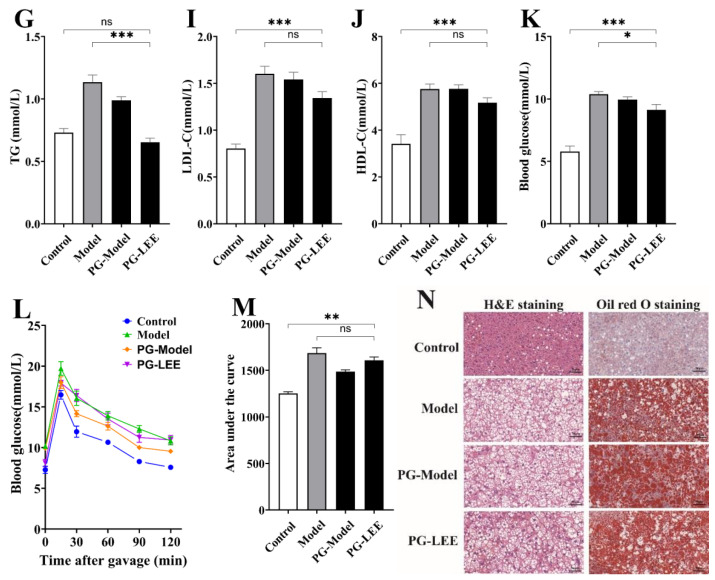
Effect of LEE on pseudo-germ-free mice with HFD. (**A**) Relative abundance at the phylum level. (**B**) Body weight gain after administration. (**C**) Liver weight. (**D**) epiWAT weight. (**E**) intBAT weight. (**F**) Food intake. Serum TC (**G**), TG (**H**), LDL-c (**I**), HDL-c (**J**), blood glucose (**K**), OGTT (**L**), area under the OGTT curve (**M**), and staining of liver tissue sections (×400) (**N**). Data are expressed as the mean ± SEM (*n* = 6). * *p* < 0.05; ** *p* < 0.01; *** *p* < 0.001; ns, not significant.

To determine whether intestinal flora is a key link in the treatment of hepatic lipid accumulation by LEE, we disrupted intestinal flora in mice using antimicrobials at the end of hepatic lipid accumulation modeling, followed by LEE intervention treatment, and tested the related indexes. Antimicrobial-treated mice exhibited a different intestinal flora structure than non-antimicrobial-treated mice, characterized by a significant increase in the relative abundance of *Protecibacteria* and *Actinobacteria*, which are thought to have higher antimicrobial tolerance, and a significant decrease in the relative abundance of *Firmicutes*, *Deferribacteres*, and *Bacteroidetes* (Figure 6A). These results suggest that antimicrobial intervention is effective in disrupting the intestinal environment of mice. From the assay index of PG-LEE mice, we found that the effect of LEE in PG mice was not significant compared with that in model mice; there were no significant differences in body weight gain, liver weight, intBAT weight, epiWAT weight, TC, TG, LDL-C, HDL-c, and glucose tolerance in PG-LEE mice (Figure 6B–M). However, there was no significant alleviation of liver fat accumulation and hepatocyte damage in PG mice after LEE treatment (Figure 6N), suggesting that the efficacy of LEE in treating liver fat accumulation disappears in PG mice and that intestinal flora is a key condition for the treatment of hepatic fat accumulation by LEE.

### 3.6. LEE Modulates HFD-Induced Abnormal Hepatic Lipid Synthesis and Inflammatory Factors

**Figure 7:**
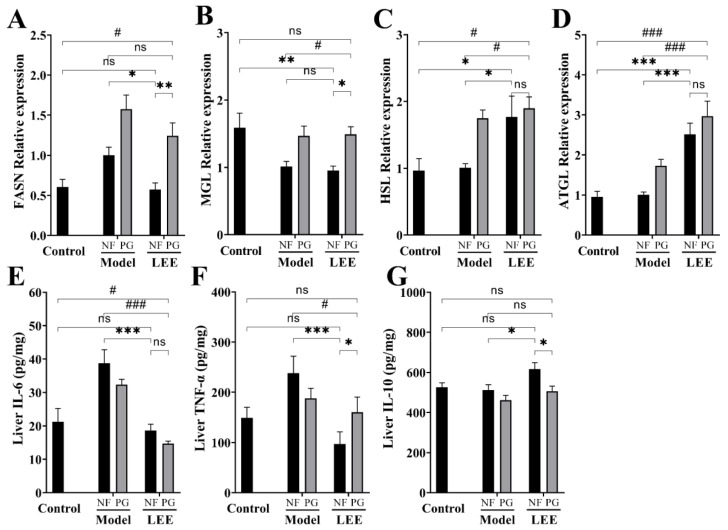
LEE administration improves HFD-induced lipid metabolism-related gene mRNA abnormal expression and inflammation in mice. Relative mRNA expression of FASN (**A**), MGL (**B**), HSL (**C**), and ATGL (**D**). Liver IL-6 (**E**), TNF-α (**F**), and IL-10 (**G**) contents. Data are expressed as the mean ± SEM (*n* = 6). * *p* < 0.05; ** *p* < 0.01; *** *p* < 0.001; # *p* < 0.05; ### *p* < 0.001; ns, not significant.

To determine the causes of the differences in hepatic lipid accumulation, we examined the lipid metabolism-related genes in the liver, including the lipase genes (ATGL, HSL and MGL), fatty acid synthase (FASN), and inflammation-related factors in the liver. Long-term HFD converts hepatic lipid metabolism to a pattern that favors lipid accumulation, which is characterized by higher fat synthesis or lower fat decomposition, and LEE administration significantly reduced this phenomenon. Compared with control mice, hepatic FASN expression was significantly increased in HFD mice (*p* < 0.01), while lipase genes (MGL, HSL, and ATGL) did not show changes favoring lipolysis (Figure 7A–D). As expected, LEE treatment shifted the hepatic lipid metabolism of HFD mice to a mode favoring lipolysis, with a significant downregulation of FASN expression and an upregulation of HSL and ATGL. Notably, we found that hepatic MGL, HSL and ATGL expression significantly increased in PG mice, but FASN was expressed in higher amounts, which may explain the failure to alleviate liver lipid accumulation in PG mice. 

ELISA kits were used to quantify the blood protein levels of TNF-α, IL-6, and IL-10 in each experimental group to study the effect of LEE on inflammation-related cytokines (Figure 7E–G). As a result, the TNF-α and IL-6 levels were found to be higher in HFD mice than those in LDF mice. The TNF-α and IL-6 levels in the liver were much lower in animals administered LEE than those in HFD mice, whereas the IL-10 levels were significantly higher. However, these efficacies were greatly compromised in PG mice (Figure 7F,G). These results suggest that LEE alleviates liver fat accumulation by improving the liver fat metabolic patterns and reducing liver inflammation in mice.

### 3.7. Impact of LEE Administration on Colonic SCFA Metabolism

**Figure 8:**
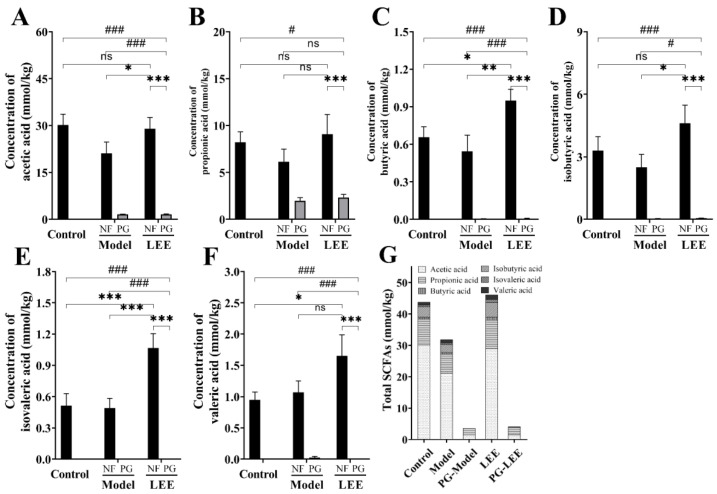
LEE increases the content of SCFAs in the colonic digesta of HFD mice. Determination of acetic acid (**A**), propionic acid (**B**), butyric acid (**C**), isobutyric acid (**D**), isovaleric acid (**E**), valeric acid (**F**), and total SCFA (**G**) content in the colonic digesta. Data are expressed as the mean ± SEM (*n* = 6). * *p* < 0.05; ** *p* < 0.01; *** *p* < 0.001; # *p* < 0.05; ### *p* < 0.001; ns, not significant.

SCFAs are mostly processed in the colon and liver, which has an impact on gut and liver function. SCFAs generated by the intestinal microbial fermentation of undigested and absorbed carbohydrates and proteins seem to play an important role in glucose homeostasis, lipid metabolism, and anti-inflammation, according to a growing body of research. The SCFA content in colonic digesta was measured to assess the impact of LEE treatment on intestinal SCFA metabolism (Figure 8A–G). SCFAs were barely detectable in the colonic digesta of mice because of the disruption of the intestinal flora in PG mice. Compared to those in control mice, HFD mice exhibited considerably lower total SCFA content in colonic digesta samples, as well as a lower content of six different SCFAs, which were mitigated by LEE treatment. Furthermore, LEE-treated HFD mice had higher concentrations of butyric and valvular acids in their colons than those in healthy mice. These findings demonstrate that LEE improves hepatic lipid metabolism and inflammation in HFD mice by modulating the production of colonic SCFAs.

### 3.8. Correlation Analysis of Feature Genera and Metabolic Parameters

**Figure 9:**
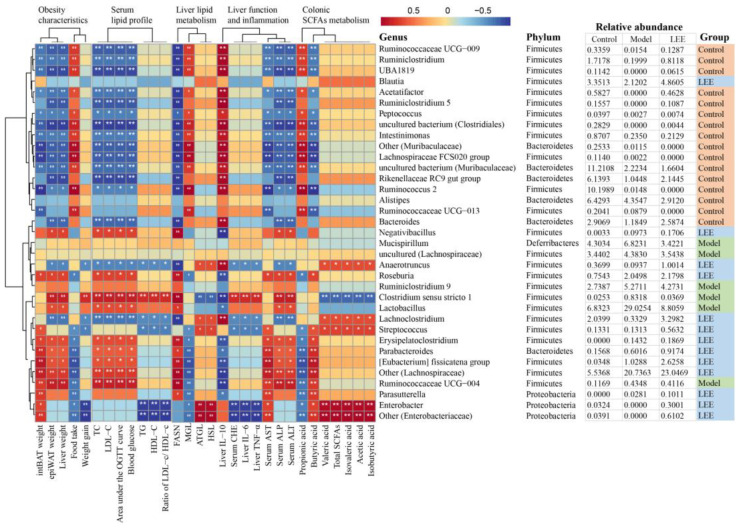
Heatmap of Spearman’s correlation between microbiota markers and liver fat accumulation-related indexes in mice altered by HFD or LEE intervention. A total of 30 microbiota markers were significantly correlated at least with one of the parameters including intBAT weight, epiWAT weight, liver weight, food intake, weight gain, TC, LDL-C, area under the OGTT curve, blood glucose, TG, HDL-C, ratio of LDL-c/ HDL-c, mRNA expression of FASN, MGL, ATGL, and HSL, liver IL-10, IL-6, and TNF-α, serum CHE, AST, ALP, and ALT, colonic digesta propionic acid concentration, butyric acid concentration, valeric acid concentration, total SCFA concentration, isovaleric, acetic and isobutyric acid concentration. The strength of the red/blue hues reflects the degree of association (blue denotes negative correlation and red denotes positive correlation). * *p* < 0.05, ** *p* < 0.01. Details (genus, phylum, and relative abundance) of the microbiota markers are shown on the right.

The dominant and characteristic genera responsible for the effect of LEE on HFD-induced hepatic lipid accumulation were determined using Spearman’s correlation analysis. Figure 9 showed that the intestinal flora was highly correlated with many liver lipid accumulation-related indicators. The feature genera of the model mice, *Clostridium sensu stricto 1* and *Lactobacillus*, were highly positively correlated with epiWAT weight, liver weight, weight gain, TC, LDL-C, area under the OGTT curve, blood glucose, TG, HDL-C, ratio of LDL-c/ HDL-c, FASN mRNA expression, serum CHE, liver IL-6, liver TNF-α, serum ALP and serum ALT, whereas they were strongly negatively correlated with liver IL-10, valeric, isovaleric, acetic and isobutyric acid, total SCFA concentration, and ATGL and HSL mRNA expression. These results suggest that these bacteria play an important role in the development of hepatic lipid accumulation diseases. LEE treatment significantly reduced the relative abundance of *Clostridium sensu stricto 1* and *Lactobacillus*, which demonstrated the value of LEE in treating liver lipid accumulation. 

Additionally, *Ruminococcaceae UCG-009*, *Ruminiclostridium*, *UBA1819*, *Acetatifactor*, *Ruminiclostridium* 5, *Rikenellaceae RC9 gut group* and *Bacteroides*, as the feature genera of control mice, were significantly negatively correlated with the development of hepatic lipid accumulation diseases, as evidenced by a decrease in twelve indicators positively associated with hepatic lipid accumulation disease and an increase in three indicators negatively associated with hepatic lipid accumulation disease. LEE intervention significantly increased the relative abundance of these genera, further demonstrating the potential of LEE for hepatic lipid accumulation therapy.

### 3.9. Effects of GL, GA and LEE on FFA-Induced HepG2 Cells

**Figure 10:**
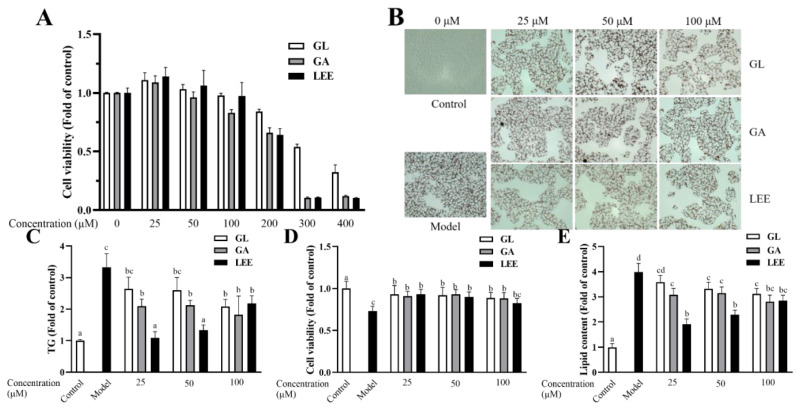
Effects of GL, GA and LEE on FFA-induced HepG2 cells. (**A**) Cell viability of HepG2 cells. (**B**) Oil Red O staining of FFA-induced HepG2 cells (400×). (**C**) Intracellular TG levels. (**D**) Cell viability of FFA-induced HepG2 cells. (**E**) Intracellular lipid levels. The results are expressed as the means ± SEM of three independent experiments. Different letters indicate significant differences in the mean values (*p* < 0.05).

GA is the main potent component of licorice, while GL is an important product of GA conversion by intestinal flora. To investigate whether GA is the only factor contributing to LEE’s efficacy in reducing liver fat, we treated HepG2 cells with 1 mM FFA and exposed them to different concentrations of GA, GL and LEE. The CCK-8 kit was used to assess the effect of different concentrations of GL, GA and LEE on HepG2 cell viability. The results showed that GL, GA and LEE treatments did not show significant toxicity on cell viability at concentrations below 100 μM, but significantly decreased cell viability above 100 μM (Figure 10A). The addition of FFA to the medium significantly reduced cell activity and increased intracellular lipid accumulation in HEPG2 cells, whereas GL, GA and LEE significantly restored the reduction in cell viability caused by FFA, while reducing intracellular lipid accumulation in HepG2 cells induced by FFA to varying degrees (Figure 10B–E). At high concentrations, there was no significant difference in the effect of GL, GA and LEE in reducing intracellular TG and lipid accumulation; however, with decreasing concentrations, the effect of LEE was significantly better than that of GL and GA, suggesting that there are other components in LEE that can effectively reduce hepatocyte fat accumulation besides GA. 

## 4. Discussion

With a rise in living standards, our dietary structures have steadily evolved toward high sugar and lipid levels, leading to an increase in obesity-related disorders [24,25]. The human gut flora has been proven to influence the pathological development of obesity and associated disorders by modulating nutritional absorption, energy consumption, fat metabolism, and immune response [26,27]. In this respect, the ingestion of functional food-derived extracts has been found to have protective effects against diet-induced gut dysbiosis [28,29,30]. Although various studies have indicated that licorice has health-promoting and anti-obesity properties in humans [15] and animal models [31,32], whether the gut flora is involved remains unclear. Thus, the efficacy of LEE in HFD-induced liver lipid accumulation and the underlying mechanisms associated with the gut microbiota were explored in this study. The findings showed that LEE administration efficiently reduced hepatic fat accumulation and associated metabolic dysfunction in mice, while significantly ameliorating the intestinal flora of HFD mice, but not in pseudo-germ-free HFD mice. These findings suggest that the gut flora is critical for the LEE treatment of liver fat accumulation and changes in liver metabolic dysfunction. 

As the largest solid organ, the liver plays a key role in human metabolism, and hepatocytes can store and deploy vast quantities of lipids as a physiological response to provide nutrition and induce changes in pathological conditions [33]. The normal liver is low in fat because it can combine fat with phosphoric acid and choline to convert it into phospholipids that are transferred to other parts of the body. However, a prolonged HFD prevents the liver from transferring fat to other organs in a timely manner, resulting in lipid accumulation in the liver. Abnormal lipid buildup in the liver has been shown to impair hepatic function and reduce the capacity of the liver to transport fat, which can exacerbate liver fat accumulation and cause a range of symptoms, such as fatty liver and liver cirrhosis [34,35]. Our findings revealed that LEE supplementation reduced liver fat accumulation in HFD mice, as evidenced by the recovery of hepatocyte morphology in H&E stained images and a significant reduction in fat content in Oil-Red O stain of LEE mice. Other indicators included reduced liver weight, TG, LDL-C and blood glucose levels, decreased expression of genes responsible for fat synthesis, and increased expression of fatty acid synthesis gene. Other studies investigating licorice extracts have also found their modulating effects on lipid metabolism, such as licochalcone A improving NAFLD, licorice root alleviating obesity traits and carbenoxolone improving liver lipid metabolism in obese mice [31,36,37,38], with the difference that we found that intestinal flora plays an essential role in the liver fat reduction efficacy of LEE. 

Accumulating evidence has revealed that HFD alters the expression levels of genes and proteins involved in hepatic fatty acid production and lipid metabolism. Therefore, gene and protein levels can be regulated to reduce liver fat accumulation [39]. Normally, hepatic fatty acid synthesis and catabolism are in dynamic balance, thus avoiding the accumulation of hepatic fat. An imbalance between fatty acid synthesis and catabolism causes fat buildup in the liver as a result of HFD. In this study, we measured the gene expression levels of enzymes related to fatty acid metabolism in the liver and showed that the expression of FASN was greatly reduced by LEE treatment, while the expression of HSL and ATGL was markedly increased. FASN is a major enzyme in fatty acid synthesis and is found in a variety of human tissues, with the highest concentrations in the liver and adipose tissues. Three primary enzymes (ATGL, HSL, and MGL) constitute the lipolytic machinery, accounting for more than 90% of the total lipase activity in the tissue [40,41]. Our study showed that the expression of lipolytic-related genes in the liver was significantly enhanced after LEE treatment, suggesting that the therapeutic effect of LEE on HFD-induced hepatic fat accumulation may be related to the reduction in lipogenesis and promotion of fatty acid catabolism. 

The intestinal flora influences health and nutritional stages through multiple mechanisms, with SCFAs being an important regulatory modality. SCFAs, consisting of acetic, propionic, butyric, and valeric acid, are produced by the intestinal microbial fermentation of nondigestible food components and play a key role in glucose homeostasis, lipid metabolism, and epithelial barrier function [20]. Dietary patterns can influence bacterial community composition and SCFAs production. The amounts of acetic, propionic, butyric acid, and total SCFAs in the intestinal digesta of HFD-mice markedly decreased in our study, which is consistent with the results in earlier results [42,43]. LEE treatment significantly increased the production of colonic SCFAs in HFD mice, and the levels of acetic acid and propionic acid in the colon returned to normal levels after LEE intervention, whereas butyric and valeric acid levels were higher than normal dietary levels, demonstrating the beneficial effect of LEE administration on the production of SCFAs in the colon. Acetic and propionic acid produced by the intestinal flora can enter the human circulation and affect the metabolic functions of tissues, such as regulating the metabolic functions of the liver and adipose tissue to alleviate hepatic steatosis and insulin resistance. Studies have observed that increased blood acetic acid concentrations are significantly correlated with decreased levels of white fat and insulin [21,44]. Butyrate, as an energetic substrate for colonic cells, plays an important role in maintaining the intestinal barrier, such as inducing the apoptosis of colonic malignant cells and regulating macrophage activity by reducing the production of proinflammatory cytokines [45]. Thus, the alterations in short-chain fatty acids in the mice colon caused by LEE could treat the detrimental effects of HFD. 

Evidence indicates that diet-induced obesity and related diseases are significantly linked to the dysregulation of the gut microbiota [46]. In our study, HFD resulted in an imbalance in the gut microbiota typical of obesity, as seen by the increased relative abundance of *Firmicutes*, reduced *Bacteroidetes*, and the greatly elevated F/B ratio, which is consistent with the findings of other studies [47,48,49]. *Firmicutes* and *Bacteroides*, the major bacterial phyla in the mammalian gut, play vital roles in controlling glucose, lipid, and bile acid metabolism [50]. Supplementation with LEE significantly altered the composition and structure of the intestinal microbiota of HFD-induced obese mice and significantly reduced the F/B ratio in HFD-fed mice, suggesting a beneficial effect on the prevention of diet-induced intestinal flora dysbiosis. Several studies have shown that modulation of the gut microbiota is responsible for the prevention of HFD-induced obesity [43,44,45]. Our study evaluated the effect of LEE administration on changes in intestinal flora at the genus level. *LEfse* analysis showed that a total of 39 OTUS (24 genera of 3 phyla) were selected as key species for HFD treatment. The heatmap of the correlation analysis showed that 27 feature genera were significantly reversed in the LEE intervention group compared with those in the HFD group. HFD resulted in a significant increase in the relative abundance of *Mucispirillum*, uncultured *(Lachnospiraceae)*, *Ruminiclostridium* 9, *Clostridium sensu stricto* 1 and *Lactobacillus*, and a decrease in *Ruminococcaceae* UCG-009, *Ruminiclostridium*, UBA1819, *Blautia*, *Acetatifactor*, *Ruminiclostridium* 5, *Rikenellaceae* RC9 gut group, *Bacteroides*, *Anaerotruncus*, *Lachnoclostridium*, *Enterobacter*, and others (*Enterobacteriaceae*), whereas intervention with LEE significantly reversed the relative abundance of these genera. Among them, *Ruminococcaceae* UCG-009, *Ruminiclostridium*, *Rikenellaceae* RC9 gut group, *Bacteroides*, *Parabacteroides* and Others *(Lachnospiraceae)* have been proven to be negatively associated with obesity and its associated diseases, whereas a high relative abundance of *Lactobacillus* is positively associated [27,41,47]. Notably, we found that the relative abundance of four feature genera, including *Mucispirillum*, *Ruminiclostridium* 9, *Clostridium sensu stricto* 1 and *Lactobacillus*, were decreased by LEE supplementation. *Clostridium sensu stricto* 1 and *Lactobacillus* were positively correlated with liver lipid accumulation. LEE intervention may significantly boost the relative abundance of 15 feature genera, of which *Ruminococcaceae* UCG-009, *Ruminiclostridium*, UBA1819, *Acetatifactor*, *Ruminiclostridium* 5, *Rikenellaceae* RC9 gut group, *Bacteroides*, *Anaerotruncus*, *Lachnoclostridium*, *Streptococcus*, *Enterobacter* and Other (*Enterobacteriaceae*) were negatively related to the development of hepatic lipid accumulation disease, indicating that these bacteria may play a key role in LEE by preventing the development of lipid accumulation in the liver. 

## 5. Conclusions

In this study, LEE was found to effectively reduce obesity and liver fat accumulation in HFD mice, and the important role of the intestinal flora in this treatment process was highlighted. We suggest that the production of SCFAs by LEE-regulated intestinal flora may be one of the mechanisms by which LEE regulates hepatic lipid metabolism and inflammation, but not exclusively. The results of cellular experiments demonstrated that the effect of GA in reducing lipid accumulation in Hepg2 cells was enhanced after being converted to GL, indicating that the intestinal flora may enhance its efficacy by converting some components in licorice. In conclusion, intestinal flora plays an essential role in the treatment of liver fat accumulation by LEE. LEE modulates the intestinal flora of HFD mice and affects the production of short-chain fatty acids, while some components of LEE may be converted into more effective components by the intestinal flora. However, we are not sure which components of LEE are converted into more effective components by the intestinal flora, and further and detailed studies are required to explore the potential conversion in the future. 

## Data Availability

All data presented in this study are available in the main body of the manuscript.
